# Association of Single Nucleotide Polymorphisms in VDR and DBP Genes with HBV-Related Hepatocellular Carcinoma Risk in a Chinese Population

**DOI:** 10.1371/journal.pone.0116026

**Published:** 2014-12-26

**Authors:** Qiliu Peng, Shi Yang, Xianjun Lao, Ruolin Li, Zhiping Chen, Jian Wang, Xue Qin, Shan Li

**Affiliations:** 1 Department of Clinical Laboratory, First Affiliated Hospital of Guangxi Medical University, Nanning, Guangxi, China; 2 Department of Medicine Research, First Affiliated Hospital of Guangxi Medical University, Nanning, Guangxi, China; 3 Department of Occupational Health and Environmental Health, School of Public Health at Guangxi Medical University, Nanning, Guangxi, China; Sanjay Gandhi Medical Institute, India

## Abstract

**Background:**

Polymorphisms of genes encoding components of the vitamin D pathway including vitamin D receptor (VDR) and vitamin D binding protein (DBP) have been widely investigated because of the complex role played by vitamin D in cancer tumorogenesis. In this study, we investigated the association between VDR and DBP gene polymorphisms and HBV-related HCC risk in a Chinese population.

**Methods:**

Study subjects were divided into three groups: 184 HBV patients with HCC, 296 HBV patients without HCC, and 180 healthy controls. The VDR rs2228570, and rs3782905 and the DBP rs7041 polymorphisms were genotyped using PCR-RFLP and the VDR rs11568820 polymorphism was genotyped by PCR-SSP, respectively. DNA sequencing was performed to validate the genotype results.

**Results:**

We found that there were significant differences in the genotype and allele frequencies of the VDR rs2228570 and DBP rs7041 polymorphisms between HBV patients with HCC and healthy controls. The rs2228570 T allele was associated with a significant increased HBV-related HCC risk as compared with the C allele. The rs2228570 TT and TT/TC genotypes were correlated with a significant increased HBV-related HCC risk when compared with the wild-type CC homozygote. Similarly, the rs7041 G allele was associated with a significant increased HBV-related HCC risk as compared with the T allele. The rs7041 GG and GG/TG genotypes were correlated with a significant increased HBV-related HCC risk when compared with the wild-type TT homozygote. However, we did not observe any significant effect of VDR rs11568820, and rs3782905 polymorphisms on HBV-related HCC risk in this population. In haplotype analysis, we also did not find any significant differences in haplotype frequencies of the VDR gene between HBV patients with HCC and the healthy controls.

**Conclusions:**

We conclude that the VDR rs2228570 and DBP rs7041 polymorphisms may contribute to increased susceptibility to HBV-related HCC in the Chinese population. Due to the marginal significance, further large and well-designed studies in diverse ethnic populations are needed to confirm our results.

## Introduction

Hepatocellular carcinoma (HCC) is the fifth most common malignancy and the third leading cause of cancer mortality worldwide [1]. The distribution of HCC is imbalance throughout the world, with particularly high incidence rates in Eastern Asia, and Sub-Saharan Africa, and China alone accounted for more than 50% of all HCC cases [1]. Major risk factors for HCC include infection with hepatitis B virus (HBV) and hepatitis C virus (HCV), dietary aflatoxin B1 exposure, and alcohol consumption [2], [3]. Overall, 75–85% of HCC patients are attributable to persistent viral infection of HBV, especially in developing countries such as China [1]. However, only a minority of lifelong chronic carriers of HBV eventually develops HCC, suggesting the importance of genetic susceptibility in HBV-related HCC.

Vitamin D is a fat soluble secosteroid which is involved in a wide variety of biological processes like bone metabolism, modulation of immune response, cell proliferation and cell differentiation [4]. Studies have reported that vitamin D implicated in inhibition of carcinogenesis by induction of differentiation, promotion of apoptosis, and inhibition of proliferation and angiogenesis [5]. Vitamin D receptor (VDR) is a crucial mediator for the cellular effects of vitamin D and additionally interacts with other cell-signaling pathways that influence cancer development [6]. It is an intracellular hormone receptor that specifically binds the biologically active form of vitamin D and interacts with specific nucleotide sequences (response elements) of target genes to produce a variety of biological effects. The VDR gene is located on chromosome 12q12–q14 and is highly polymorphic [7]. Many single-nucleotide polymorphisms (SNPs) have been identified and genetic variations in the VDR gene may phenotypically appear as interindividual variations in limiting rates of vitamin D synthesis in the skin, hydroxylation in the liver and kidney, transport, metabolism and degradation that would ultimately influence the anti-tumor effect of vitamin D. Moreover, the VDR gene variants have been reported to associate with increased risk of cancers including breast [8], prostate [9], and colorectal [10].

Vitamin D binding protein (DBP) is the primary transport protein of 25(OH)D in circulation, with approximately 88% of 25(OH)D being bound to DBP [11]. In addition to being a carrier for vitamin D metabolites, DBP also has anti-inflammatory and immunoregulatory functions, and has been identified playing a role in several chronic disease including cancers [12]. The gene for the DBP is also a logical candidate in the vitamin D pathway because of its major function in transporting vitamin D metabolites in the circulation. Variants in this gene have been shown to alter plasma concentrations of 25-OHD [13]. In addition, the variants in the DBP gene have been implicated in cancers such as breast [14] and prostate [15].

In light of the important biological function of VDR and DBP genetic polymorphisms in cancer development and progression, we hypothesized that genetic variants of the VDR and DBP genes were associated with increased susceptibility to HBV-related HCC. VDR rs11568820, rs2228570, and rs3782905 and DBP rs7041 polymorphisms are the most common studied SNPs which have been found modulate the expressions of vitamin D and play critical roles in carcinogenesis [12], [16], [17], [18]. Therefore, we performed a case–control study to investigate the association between VDR rs11568820, rs2228570, and rs3782905 and DBP rs7041 polymorphisms and HBV-related HCC susceptibility in a Chinese population.

## Materials and Methods

### Study population

This is a hospital-based case-control study performed in a total of 660 subjects, including 184 HBV patients with HCC, 296 HBV patients without HCC, and 180 healthy controls. All study subjects were consecutively recruited between July 2013 and November 2013 from the First Affiliated Hospital of Guangxi Medical University, Guangxi, China without any restrictions on age, gender, drinking and smoking. The HBV patients with HCC were diagnosed based on pathological findings combined with at least one positive HCC image on computed tomography (CT) or magnetic resonance imaging (MRI), sometimes combined with serum AFP analysis (>400 ng/ml). All patients with HBV infection selected for this study (including HCC and non-HCC patients) were further confirmed by being HBsAg (hepatitis B surface antigen) positive, HbcAb (hepatitis B virus core antibody) positive and HBeAg (hepatitis B e antigen) or HBeAb (hepatitis B e antibody) positive for at least 6 months. The presence of HCC was excluded from the non-HCC patients and healthy controls by magnetic resonance imaging (MRI), computed tomography (CT), ultrasonography, histology, and other laboratory tests. Moreover, patients with positive laboratory tests for hepatitis C virus (HCV; anti-HCV and/or HCV-RNA), human immunodeficiency virus (HIV), alcoholic liver disease, or suspected autoimmune diseases with antinuclear antibody titer greater than 1∶160 were excluded from this study. A sample of approximately 2–4 ml venous blood was collected from each participant. The following laboratory parameters were obtained for each participant at the time of whole-blood collection: serum total bilirubin (T-Bil), direct bilirubin (D-Bil), aspartate aminotransferase (AST), alanine aminotransferase (ALT), albumin (ALB), gamma-glutamyltransferase (GGT), alpha fetoprotein (AFP), and HBV-DNA. In addition, HBsAg, HBsAb, HbcAb, HBeAg, and HBeAb were also collected. All participants were exclusively enrolled from Guangxi district with written informed consent and the study was performed with the approval of the Ethics Committee of the First Affiliated Hospital of Guangxi Medical University.

### Genomic DNA preparation and genotyping

Genomic DNA was extracted from 2 mL of peripheral blood using the standard phenol–chloroform method, dissolved in 300 µL of Tris–HCl buffer (10 mmol/L, pH 8.0) containing 1 mmol/L ethylenediaminetetraacetic acid (EDTA), and stored at −80°C until use. The SNPs of VDR rs2228570, and rs3782905 and DBP rs7041 were detected using polymerase chain reaction–restriction fragment length polymorphism (PCR-RFLP), and the VDR rs11568820 polymorphism was genotyped by polymerase chain reaction-sequence specific primer (PCR-SSP). Primer sequences, annealing temperatures, restriction enzymes, and product size in genotype analysis are shown in Table 1. To control the quality of genotyping, 50% of samples were repeated.

**Table 1:** Primer sequence and reaction condition.

SNPs	Primer sequence	Annealing temperature	Restriction enzyme	Product size (bp)
rs2228570	F: 5′-AGCTGGCCCTGGCACTGACTCTGGCTCT-3′	60.0°C	BseG I	CC: 267 bp
	R: 5′-ATGGAAACACCTTGCTTCTTCTCCCTC-3′			CT: 267+204+63 bp
				TT: 204+63 bp
rs3782905	F: 5′-AAGACATGGTGTCTGCTTCA-3′	56.0°C	HpyF3 I	CC: 223+81 bp
	R: 5′-GGTTAGATCGATATGTTTGA-3′			CG: 304+223+81 bp
				GG: 304 bp
rs7041	F: 5′-AAATAATGAGCAAATGAAAGAAGAC-3′	56.0°C	BsuR I	TT: 482 bp
	R: 5′-CAATAACAGCAAAGAAATGAGTAGA-3′			TG: 482+298+184 bp
				GG: 298+184 bp
rs11568820	F1: 5′-AGGATAGAGAAAATAATAGAAAACATT-3′	45.0°C		GG: 297+110 bp
	R1: 5′-AACCCATAATAAGAAATAAGTTTTTAC-3′			GA: 297+235+110 bp
	F2: 5′-TCCTGAGTAAACTAGGTCACAA-3′			AA: 297+235 bp
	R2: 5′-ACGTTAAGTTCAGAAAGATTAATTC-3′			

**Figure 1:**
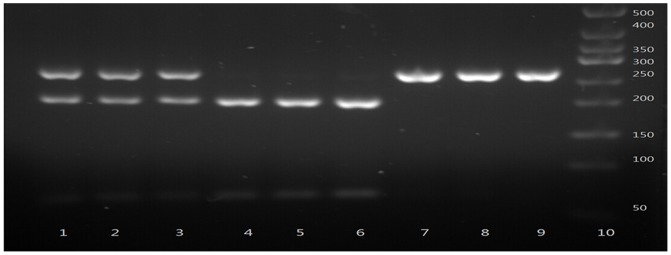
Polymerase chain reaction–restriction fragment length polymorphism assay for analyzing VDR rs2228570 polymorphism. Lane 10 shows marker; Lanes 1, 2 and 3 show rs2228570 TC genotype; lanes 4, 5 and 6 show rs2228570 TT genotype; lanes 7, 8 and 9 show rs2228570 CC genotypes.

**Figure 2:**
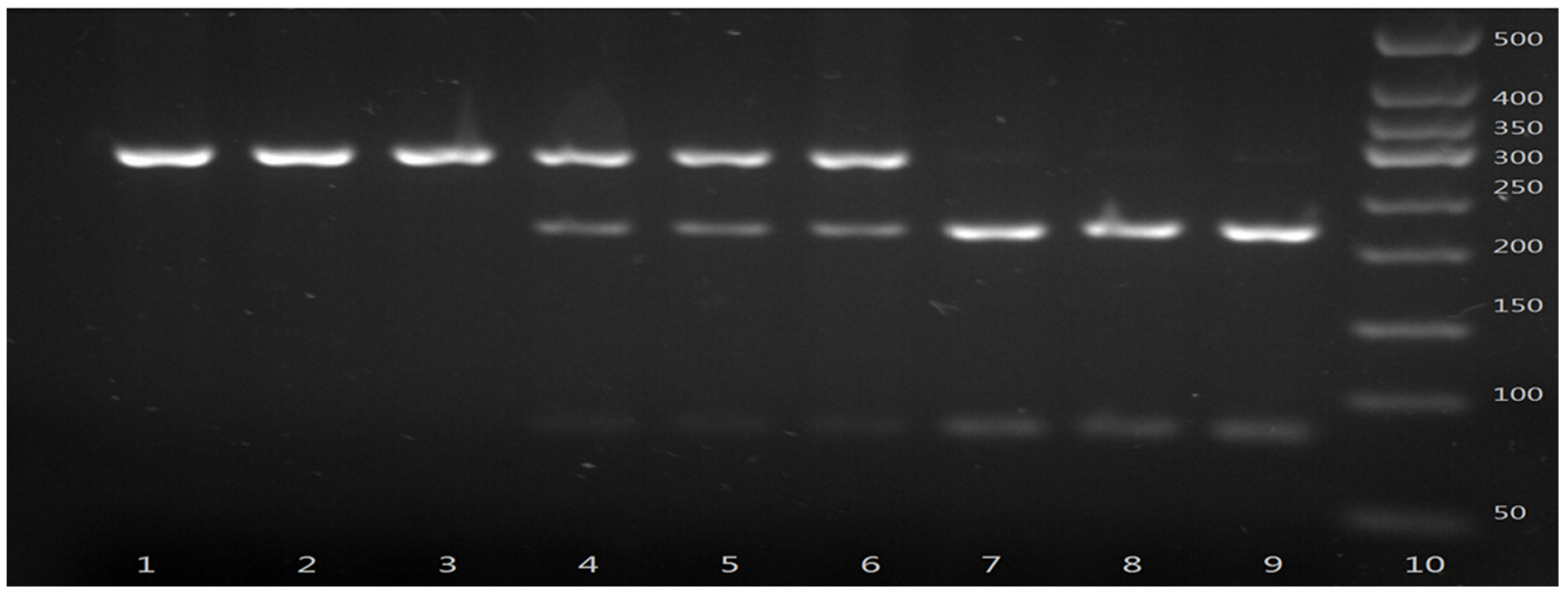
Polymerase chain reaction–restriction fragment length polymorphism assay for analyzing VDR rs3782905 polymorphism. Lane 10 shows marker; Lanes 1, 2 and 3 show rs3782905 GG genotype; lanes 4, 5 and 6 show rs3782905 CG genotype; lanes 7, 8 and 9 show rs3782905 CC genotypes.

**Figure 3:**
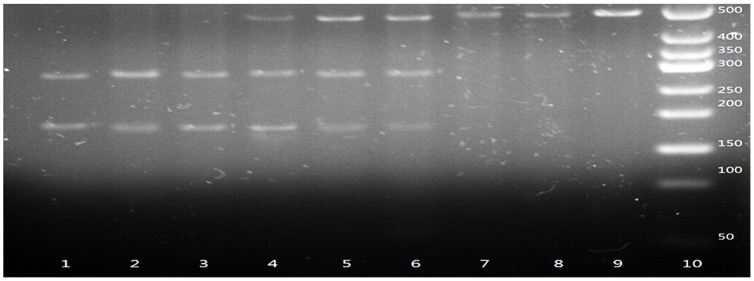
Polymerase chain reaction–restriction fragment length polymorphism assay for analyzing DBP rs7041 polymorphism. Lane 10 shows marker; Lanes 1, 2 and 3 show rs7041 GG genotype; lanes 4, 5 and 6 show rs7041 TG genotype; lanes 7, 8 and 9 show rs7041 TT genotype.

The gene fragments containing the polymorphic sites were amplified by PCR. PCR was performed in a total volume of 25 µL reaction mixture containing 1 U DNA Taq polymerase (Shanghai Biocolor, Shanghai, China), 1× buffer, 50 ng genomic DNA, 1.5 mmol/L MgCl2, 0.2 µmol/L primers, and 0.08 mmol/L dNTPs. The PCR cycles were as follows: 94°C for 5 min, 30 cycles of denaturing at 94 °C for 45 s, annealing at the indicated temperature for 45 s, extension at 72 °C for 45 s, and a final extension at 72 °C for 5 min in a Perkin-Elmer thermocycler (2700, Applied Biosystems, Foster City, CA, USA). After amplification, the PCR products were identified under ultraviolet light after electrophoresis for 15 min with 120 V in 2.5% agarose gel stained with ethidium bromide (EB). Then the PCR products were digested by allele-specific restriction enzymes overnight at 37°C. Cleaved DNA fragments were identified by ultraviolet light after electrophoresis in 2.5% agarose gel stained by EB (Figure 1–3).

**Figure 4:**
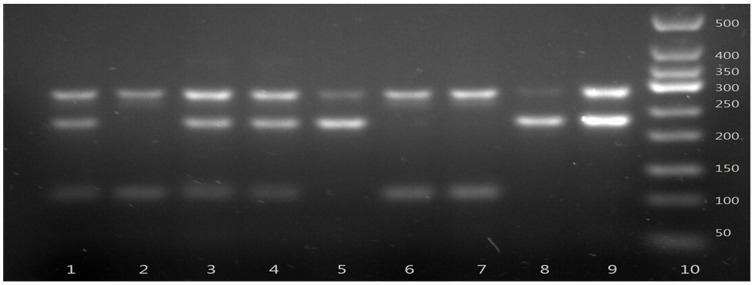
Polymerase chain reaction-sequence specific primer assay for analyzing the VDR rs11568820 polymorphism. Lane 10 shows marker; Lanes 1, 3 and 4 show rs11568820 AG genotype; Lanes 2, 6 and 7 show rs11568820 GG genotype; Lanes 5, 8 and 9 show rs11568820 AA genotype.

**Figure 5:**
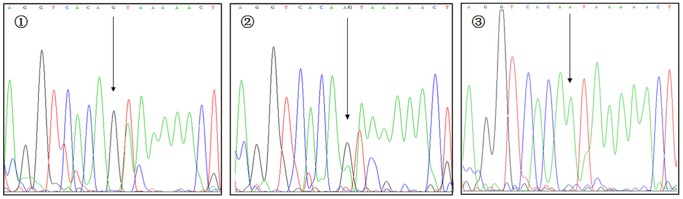
Sequencing map for genotypes of VDR rs11568820 polymorphism. The arrows in 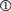
–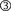 show GG, AG, and AA genotypes, respectively

**Figure 6:**
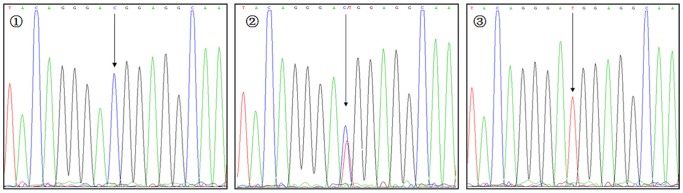
Sequencing map for genotypes of VDR rs2228570 polymorphism. The arrows in 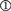
–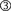 show CC, TC, and TT genotypes, respectively

**Figure 7:**
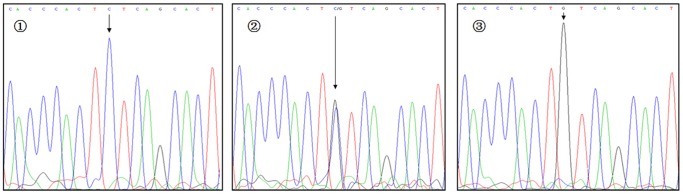
Sequencing map for genotypes of VDR rs3782905 polymorphism. The arrows in 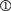
–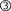 show CC, GC, and GG genotypes, respectively

**Figure 8:**
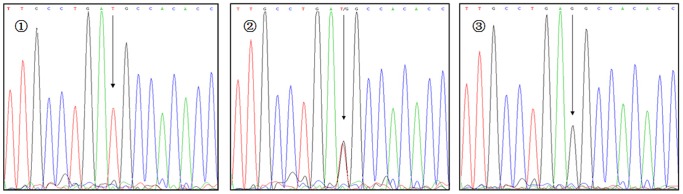
Sequencing map for genotypes of VDBP rs7041 polymorphism. The arrows in 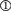
–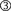 show TT, TG, and GG genotypes, respectively

VDR rs11568820 polymorphism was detected using forward primers designed with the last nucleotide complementary to the allelic variant substitution base as shown in Table 1. PCR products were illuminated by ultraviolet light after electrophoresis in 2.5% agarose gel stained by EB (Figure 4). The allelic type was determined according to the presence or absence of the desired length of PCR products (235 bp). In order to avoid pseudopositive or pseudonegative results in SSP-PCR, positive and negative controls were included each time. To confirm the genotyping results, 10% of PCR-amplified DNA samples in each group (totally 66 specimens) were examined by DNA sequencing, and the results were 100% concordant (Figure 5–8.).

### Statistical analysis

Continuous variables were expressed as mean±standard deviation (SD). Differences in demographic characteristics and laboratory parameters such as T-Bil, D-Bil, AST, ALT, ALB, GGT, AFP, and HBV-DNA were compared among groups with one-way ANOVA (for continuous variables) and χ^2^ test (for categorical variables). The Hardy–Weinberg equilibrium (HWE) was evaluated with a goodness of fit χ^2^-test with one degree of freedom to compare the observed genotype frequencies among the subjects with the expected genotype frequencies. Genotype and allele frequencies of VDR rs11568820, rs2228570, and rs3782905 and DBP rs7041 polymorphisms were compared among various groups using the χ^2^ test and fisher's exact test when appropriate, and the odds ratios (ORs) and 95% confidence intervals (CIs) were calculated by binary logistic regression adjusted for age, gender, smoking, and drinking to assess the relative risk conferred by a particular allele and genotype. To assess the effect of the genotypes carrying the variant allele we ran analysis under the dominant model. The linkage disequilibrium (LD) among the three polymorphisms in the VDR gene was quantified using Shi's standardized coefficient D′ [19]. The haplotypes and their frequencies were estimated based on a Bayesian algorithm using the Phase program [20]. All tests were two-sided with a significance level of P<0.05. All statistical analyses were performed using SPSS statistical software package version 13.0 (SPSS, Inc., Chicago, IL, USA).

## Results

### Demographic and clinical characteristics

The demographic and laboratory parameters of all subjects enrolled in this study were summarized in Table 2. The demographic data included age, gender, smoking, and drinking, and the laboratory parameters included T-Bil, D-Bil, AST, ALT, ALB, GGT, AFP, and HBV-DNA. Briefly, the healthy controls and the HBV patients without HCC were significantly younger than the HBV patients with HCC (P<0.001). The HBV patients with HCC had a higher proportion of smokers and drinkers compared with the other two groups (P<0.05). In addition, the HBV patients with HCC and the other two groups without HCC (healthy controls and HBV patients without HCC) had statistical significant different laboratory results for T-Bil, D-Bil, AST, ALT, ALB, GGT, AFP (P<0.001), and HBV-DNA (P<0.05).

**Table 2:** Demographic and laboratory parameters of the subjects included in the study.

Groups	Healthy controls (n = 180)	HBV patients without HCC (n = 296)	HBV patients with HCC (n = 184)	P value
Demographic parameters				
Gender (M/F)	154/26	247/49	163/21	0.299
Age (years) (mean±SD)	38.13±11.93	42.47±12.84	49.02±11.42	<0.001
Smoking, n (%)				
Yes	134 (74.4)	227 (76.7)	155 (84.2)	0.023
No	46 (25.6)	69 (23.3)	29 (15.8)	
Drinking, n (%)				
Yes	147 (81.7)	245 (82.8)	166 (90.2)	0.041
No	33 (18.3)	51 (17.2)	18 (9.8)	
Laboratory parameters (mean±SD)				
T-Bil^a^ (µmol/L)	9.71±3.36	13.87±5.07	31.47±11.89	<0.001
D-Bil^a^ (µmol/L)	3.67±0.86	5.43±2.24	11.84±3.59	<0.001
AST^b^ (IU/L)	13.74±2.19	44.23±11.94	59.49±17.97	<0.001
ALT^b^ (IU/L)	11.57±2.26	51.97±14.19	67.84±19.59	<0.001
ALB^b^ (g/L)	49.23±17.31	38.59±14.22	26.39±11.43	<0.001
GGT^b^ (IU/L)	19.47±4.64	56.42±16.37	74.67±19.59	<0.001
AFP^b^ (ng/ml)	9.12±0.67	11.43±2.29	978.34±547.49	<0.001
HBV-DNA, log_10_ (copies/ml)	No data	4.27±1.42	3.96±1.21	0.014

ALB serum albumin level, T-Bil serum total bilirubin level, ALT serum alanine aminotransferase level, AST serum aspartate aminotransferase level, GGT serum gamma-glutamyltransferase level, AFP serum alpha fetoprotein level.

a Significant difference exists between the HCC group and non-HCC groups (P<0.001).

b Significant difference exists between each group (P<0.001).

### Genotype and allele distributions of the VDR and DBP polymorphisms

The genotype and allele distributions of VDR and DBP polymorphisms in 184 HBV patients with HCC, 296 HBV patients without HCC, and 180 healthy controls were determined using PCR-RFLP and PCR-SSP methods. The genotype and allele distributions of VDR rs11568820, rs2228570, and rs3782905 and DBP rs7041 polymorphisms were shown in Table 3. The genotype frequencies for the 4 SNPs in healthy controls were consistent with the study of Asian populations (Han Chinese in Beijing) in the International HapMap project (http://hapmap.ncbi.nlm.nih.gov/cgi-perl/gbrowse/hapmap24_B36/). Furthermore, the genotype frequencies for the 4 SNPs were all consistent with HWE in the healthy controls (P = 0.238 for rs11568820, P = 0.351 for rs2228570, P = 0.650 for rs3782905, and P = 0.819 for rs7041, respectively).

**Table 3:** Association analysis of VDR and DBP gene polymorphisms in HBV-related patients and healthy controls.

SNPs	Healthy controls (n = 180)	HBV patients without HCC (n = 296)	HBV patients with HCC (n = 184)	HBV patients without HCC versus healthy controls		HBV patients with HCC versus healthy controls		HBV patients with HCC versus HBV patients without HCC	
				OR (95% CI)†	p value†	OR (95% CI) †	p value†	OR (95% CI) †	p value†
VDR									
rs11568820									
GG	61 (33.9)	126 (42.6)	63 (34.2)	1.00 (Ref)		1.00 (Ref)		1.00 (Ref)	
AG	94 (52.2)	130 (43.9)	92 (50.0)	0.67 (0.45–1.00)	0.052	0.92 (0.59–1.52)	0.817	1.42 (0.95–2.12)	0.091
AA	25 (13.9)	40 (13.5)	29 (15.8)	0.78 (0.43–1.39)	0.392	1.05 (0.51–2.16)	0.722	1.45 (0.82–2.55)	0.197
AG+AA				0.69 (0.47–1.02)	0.060	0.93 (0.61–1.41)	0.661	1.42 (0.97–2.09)	0.069
G allele	216 (60.0)	382 (64.5)	218 (59.2)	1.00 (Ref)		1.00 (Ref)		1.00 (Ref)	
A allele	144 (40.0)	210 (35.5)	150 (40.8)	0.83 (0.63–1.08)	0.161	1.00 (0.72–1.39)	0.834	1.25 (0.96–1.64)	1.000
rs2228570									
CC	53 (29.4)	67 (22.6)	40 (21.7)	1.00 (Ref)		1.00 (Ref)		1.00 (Ref)	
TC	84 (46.7)	152 (52.4)	90 (48.9)	1.43 (0.91–2.24)	0.116	1.73 (0.98–3.04)	0.057	0.99 (0.62–1.59)	0.973
TT	43 (23.9)	77 (26.0)	54 (29.3)	1.42 (0.84–2.38)	0.118	2.15 (1.13–4.08)	0.032	1.18 (0.70–1.98)	0.547
TC+TT				1.43 (0.94–2.17)	0.097	1.82 (1.04–3.27)	0.039	1.05 (0.68–1.64)	0.819
C allele	190 (52.8)	286 (48.3)	170 (46.2)	1.00 (Ref)		1.00 (Ref)		1.00 (Ref)	
T allele	170 (47.2)	306 (51.7)	198 (53.8)	1.20 (0.92–1.55)	0.181	1.49 (1.08–2.06)	0.022	1.09 (0.84–1.41)	0.523
rs3782905									
CC	125 (69.4)	204 (68.9)	124 (67.4)	1.00 (Ref)		1.00 (Ref)		1.00 (Ref)	
CG	51 (28.3)	81 (27.4)	58 (31.5)	0.97 (0.64–1.47)	0.898	1.09 (0.66–1.80)	0.552	1.18 (0.79–1.77)	0.427
GG	4 (2.2)	11 (3.7)	2 (1.1)	1.69 (0.53–5.41)	0.376	0.23 (0.04–1.58)	0.425	0.30 (0.07–1.37)	1.000
CG+GG				1.03 (0.69–1.53)	0.904	1.02 (0.54–1.64)	0.474	1.07 (0.72–1.59)	0.726
C allele	301 (83.6)	489 (82.6)	306 (83.2)	1.00 (Ref)		1.00 (Ref)		1.00 (Ref)	
G allele	59 (16.4)	103 (17.4)	62 (16.8)	1.08 (0.76–1.53)	0.688	0.93 (0.60–1.43)	0.868	0.96 (0.68–1.36)	0.826
DBP									
rs7041									
TT	94 (52.2)	141 (47.6)	87 (47.3)	1.00 (Ref)		1.00 (Ref)		1.00 (Ref)	
TG	73 (40.6)	119 (40.2)	78 (42.4)	1.09 (0.74–1.61)	0.677	1.36 (0.84–2.21)	0.315	1.06 (0.72–1.57)	0.762
GG	13 (7.2)	36 (12.2)	19 (10.3)	1.85 (0.93–3.67)	0.077	2.58 (1.12–5.92)	0.023	0.86 (0.46–1.59)	0.619
TG+GG				1.20 (0.83–1.74)	0.332	1.74 (1.02–3.84)	0.039	1.01 (0.70–1.47)	0.940
T allele	261 (72.5)	401 (67.7)	252 (68.5)	1.00 (Ref)		1.00 (Ref)		1.00 (Ref)	
G allele	99 (27.5)	191 (32.3)	116 (31.5)	1.26 (0.94–1.68)	0.121	1.50 (1.06–2.14)	0.034	0.97 (0.73–1.28)	0.811

† Adjusted for age, sex, smoking and drinking. OR, odds ratio; CI, confidence interval.

### HBV patients without HCC compare with healthy controls

Genotype and allele frequencies of the VDR rs11568820, rs2228570, and rs3782905 and DBP rs7041 polymorphisms between HBV patients without HCC and healthy controls are presented in Table 3. There were no significant differences in the genotype and allele frequencies of VDR rs11568820, rs2228570, and rs3782905 and DBP rs7041 polymorphisms between the HBV patients without HCC and healthy controls. Binary logistic regression analyses adjusted for age, gender, smoking and drinking also did not reveal any significant difference between the VDR rs11568820, rs2228570, and rs3782905 and DBP rs7041 polymorphisms and CHB risk.

### HBV patients with HCC compare with HBV patients without HCC

Genotype and allele frequencies of the VDR rs11568820, rs2228570, and rs3782905 and DBP rs7041 polymorphisms between HBV patients with HCC and HBV patients without HCC are shown in Table 3. We also did not observe any significant differences in genotype and allele frequencies of the VDR rs11568820, rs2228570, and rs3782905 and DBP rs7041 polymorphisms between HBV patients with HCC and HBV patients without HCC. Similarly, binary logistic regression analyses adjusted for age, gender, smoking and drinking also did not reveal any significant difference between the VDR rs11568820, rs2228570, and rs3782905 and DBP rs7041 polymorphisms and HCC risk.

### HBV patients with HCC compare with healthy controls

Genotype and allele frequencies of the VDR rs11568820, rs2228570, and rs3782905 and DBP rs7041 polymorphisms between HBV patients with HCC and healthy controls are summarized in Table 3. The results showed that there were no significant differences in the genotype and allele frequencies of the VDR rs11568820 and rs3782905 polymorphisms between the HBV patients with HCC and healthy controls. Binary logistic regression analyses adjusted for age, gender, smoking and drinking also did not reveal any significant difference between VDR rs11568820 and rs3782905 polymorphisms and HCC risk. However, with respect to VDR rs2228570 polymorphism, the frequencies of the CC, TC and TT genotypes were 29.4, 46.7 and 23.9% in healthy controls and were 21.7, 48.9 and 29.3% in HBV patients with HCC, respectively. The frequencies of the C and T alleles were 52.8 and 47.2% in healthy controls and were 46.2 and 53.8% in HBV patients with HCC, respectively. The results indicated that there were significant differences in the genotype and allele frequencies of the VDR rs2228570 polymorphism between HBV patients with HCC and healthy controls. Binary logistic regression analyses adjusted for age, gender, smoking and drinking showed that the rs2228570 T allele was associated with a significant increased HCC risk as compared with the C allele (adjusted OR = 1.49, 95%CI, 1.08–2.06, P = 0.022). The rs2228570 TT and the rs2228570 TT/TC genotypes were correlated with a significant increased HCC risk when compared with the wild-type CC homozygote (adjusted OR = 2.15, 95% CI, 1.13–4.08, P = 0.032 for TT, and adjusted OR = 1.82, 95% CI, 1.04–3.27, P = 0.039 for TT/TC). With respect to DBP rs7041 polymorphism, the frequencies of the TT, TG and GG genotypes were 52.2, 40.6 and 7.2% in healthy controls and were 47.3, 42.4 and 10.3% in HBV patients with HCC, respectively. The frequencies of the T and G alleles were 72.5 and 27.5% in healthy controls and were 68.5 and 31.5% in HBV patients with HCC, respectively. We observed that there were also significant differences in the genotype and allele frequencies of the DBP rs7041 polymorphism between HBV patients with HCC and healthy controls. Binary logistic regression analyses adjusted for age, gender, smoking and drinking revealed that the rs7041 G allele was associated with a significant increased HCC risk as compared with the T allele (adjusted OR = 1.50, 95% CI, 1.06–2.14, P = 0.034). The rs7041 GG and the rs7041 TG/GG genotypes were correlated with a significant increased HCC risk when compared with the wild-type CC homozygote (adjusted OR = 2.58, 95% CI, 1.12–5.92, P = 0.023 for TT, and adjusted OR = 1.74, 95% CI, 1.02–3.84, P = 0.039 for TG/GG).

### Haplotype distributions of VDR gene in HBV patients with HCC and healthy controls

Haplotype analyses were performed in the VDR gene between HBV patients with HCC and healthy controls using the SHEsis software and the possible eight haplotype frequencies are shown in Table 4. Overall, strong linkage disequilibrium was observed between the rs2228570 C allele and the rs11568820 G allele (D' = 0.943) and rs3782905 C allele (D' = 0.962). Major GCC haplotype accounted for 25.7% and 22.5% of the eight haplotypes in both the HBV patients with HCC and healthy controls, respectively. However, the haplotype frequencies of the VDR gene in HBV patients with HCC were not significantly different from that in healthy controls (P>0.05).

**Table 4:** Haplotype distributions of VDR gene in HBV-related HCC patients and healthy controls.

Haplotypes	HBV-related HCC, 2n = 368 (%)	Healthy controls, 2n = 360 (%)	?^2^	*p*	OR (95%CI)
GCC	94.52 (25.7)	81.13 (22.5)	1.312	0.252	1.220 (0.868–1.716)
ACC	38.03 (10.3)	56.99 (15.8)	6.059	0.114	0.712 (0.423–1.106)
ACT	84.12 (22.9)	69.46 (19.3)	1.737	0.187	1.272 (0.889–1.819)
AGC	23.61 (6.4)	17.53 (4.9)	0.942	0.332	1.369 (0.725–2.585)
AGT	1.23 (0.3)	0.03 (0.0)	-	-	-
GCT	92.32 (25.1)	93.43 (26.0)	0.012	0.911	0.981 (0.703–1.370)
GGC	19.83 (5.4)	34.36 (9.5)	6.506	0.111	0.643 (0.348–1.096)
GGT	14.33 (3.9)	7.09 (2.0)	2.505	0.114	2.059 (0.827–5.126)

## Discussion

Hepatocellular carcinoma (HCC) is one of the most common causes of cancer mortality worldwide [1]. A better understanding of the mechanism involved in HCC is essential for us to develop new preventions and improve the existing therapies. It has been well accepted that HCC is a complex disease for which the underlying causes remain unclear. In this study, we investigated the VDR and DBP gene polymorphisms and determined whether the genetic factors are related to the occurrence of HBV-related HCC in a Chinese population. The results revealed that the VDR rs2228570 and DBP rs7041 polymorphisms were both significantly associated with HBV-related HCC. The rs2228570 T allele was associated with a significant increased HCC risk as compared with the C allele. The rs2228570 TT and the rs2228570 TT/TC genotypes were correlated with a significant increased HCC risk when compared with the wild-type CC homozygote. Similarly, the rs7041 G allele was associated with a significant increased HCC risk as compared with the T allele. The rs7041 GG and the rs7041 TG/GG genotypes were correlated with a significant increased HCC risk when compared with the wild-type CC homozygote. However, in haplotype analyses, we did not observe any significant association between VDR gene haplotypes and HBV-related HCC risk. Our results suggest that the VDR rs2228570 and DBP rs7041 polymorphisms may contribute to increased susceptibility to HBV-related HCC in the Chinese population. However, because the significant differences were only found in HBV patients with HCC versus healthy controls comparison but not in HBV patients with HCC versus HBV patients without HCC comparison, the results should be interpreted with caution.

Vitamin D is a steroid hormone with potent antitumor effects. Several investigations have demonstrated that 1, 25-dihydroxy vitamin D3 [1,25(OH)2D3], the active form of vitamin D, plays an essential role in the development of cancers by regulating the expression of tumor-related genes or mediating inhibition of growth of cancer cells [21], [22]. The VDR, a nuclear transcription factor that belongs to the steroid hormone receptor family, is an important regulator of the vitamin D pathway. It was reported that the VDR mediates gene expression of vitamin D [23] and most of the effects of active vitamin D in a wide variety of tissues [24]. In addition, in vitro studies have demonstrated that the VDR plays a critical role in regulating cell proliferation, differentiation and induction of apoptosis [25], [26]. A recent study by Beilfuss et al. [27] demonstrated that the polymorphisms in the VDR gene influenced VDR expressions and production and involved in the fibrotic processes in human hepatic stellate cells. The rs2228570 polymorphism is located in the 5′ end of the VDR gene. This polymorphism results in an alternative transcription initiation site, leading to a protein variant with three additional amino acids at the amino terminus [28]. The change in the VDR protein structure may lead to altered biological functions of vitamin D and involved in carcinogenesis [29]. Shafia et al. [30] reported that the VDR rs2228570 polymorphism was involved in the increased susceptibility to development and progression in multiple myeloma in an ethnic Kashmiri population, the homozygous (TT) genotype was associated with higher risk for developing multiple myeloma. Sarkissyan et al. [31] found that genetic variation of VDR rs2228570 influence CRC risk, particularly in African American cohorts. Similarly, Mohapatra et al. [4] reported that the VDR rs2228570 polymorphism was associated with an increased epithelial ovarian cancer risk in Indian population. In the present study, we found that the VDR rs2228570 polymorphism contributed to increased susceptibility to HBV-related HCC in Chinese population, which was consistent with the findings above.

DBP is a multifunctional protein known for its role in the transport of vitamin D metabolites. Almost all the 25(OH) D3 and active hormone 1, 25(OH)2D3 exist in the circulation bound to serum DBP [11]. It was reported that DBP regulate the half-life of 25(OH) D3 in the circulation through stabilization of the hormone and helps maintain serum vitamin D levels through reuptake by proximal tubule cells in the kidney [32]. In addition, DBP also has anti-inflammatory and immunoregulatory functions, and has been identified play important roles in several chronic disease including cancers [33]. The gene for the DBP is highly polymorphic, with more than 120 known variants and with different frequency distributions in diverse populations [34]. The rs7041 is one of the most common SNPs in the DBP gene which has been shown conversely associated with plasma concentrations of 25(OH)D3 [13], [35]. Zhou et al. [36] reported that the DBP rs7041 polymorphism was correlated with increased gastrointestinal cancer susceptibility in a Chinese population. Anderson et al. [14] demonstrated that the DBP rs7041 polymorphism interacts with Vitamin D exposure and contributes to increased breast cancer risk among a Caucasian Women in Canada. However, Poynter et al. [37] did not found a significant association between DBP rs7041 polymorphism and colorectal cancer in an American population. In this study, we observed that the DBP rs7041 polymorphism contributed to an increased HBV-related HCC risk in Chinese population. The inconsistent results may be attributed to the diverse physiological and pathophysiological effects of DBP rs7041 polymorphism on different ethnicities and different tissues.

Haplotypes are a group of closely relevant genetic markers present on one chromosome which tend to be inherited together and more frequent than expected by chance in a block pattern due to the presence of linkage disequilibrium [38]. In the present study, strong linkage disequilibrium was observed between the rs2228570 C allele and the rs11568820 G allele (D' = 0.943) and rs3782905 C allele (D' = 0.962) in the VDR gene, which was consistent with the results from Kaabachi et al. [39], suggesting that the three SNPs in the VDR gene may interact with each other and influence cancer risk. However, in the haplotype analysis, we did not observe any significant association between haplotypes and HBV-related HCC risk. The reason may be that the relatively small sample size in each haplotype has insufficient statistical power to detect a slight effect or may have generated a fluctuated risk estimate. Therefore, further studies with large sample size in diverse ethnicities are still needed to validate our findings.

Some limitations of this study need to be addressed. First of all, the study subjects were from hospitals and may not be representative of the general population. However, we applied rigorous epidemiological design by limiting factors of selecting subjects and used further statistical adjustment to minimize the potential biases. Second, the study population was restricted to Guangxi district, so it does not permit extrapolation of the results to other populations in other areas. Third, the sample size of this study is relatively small, especially for haplotype analysis, which may not have enough statistical power to explore the true association. Therefore, large population-based prospective studies with ethnically diverse populations are warranted to further elucidate the impact of VDR and DBP SNPs on HCC susceptibility.

In conclusion, our study suggested that the VDR rs2228570 and DBP rs7041 polymorphisms were associated with a significantly increased risk of HBV-related HCC in this Chinese population. This is the first report that the potentially functional SNP in the promoter region of VDR and DBP may contribute to HBV-related HCC susceptibility in the Chinese population. Due to the marginal significance, larger well-designed epidemiological studies with ethnically diverse populations and functional evaluations are warranted to confirm our findings.
